# Technical Note: The use of DirectDensity^TM^ and dual‐energy CT in the radiation oncology clinic

**DOI:** 10.1002/acm2.12546

**Published:** 2019-03-09

**Authors:** Geoff Nelson, Vadim Pigrish, Vikren Sarkar, Fan‐Chi Su, Bill Salter

**Affiliations:** ^1^ Department of Radiation Oncology University of Utah Salt Lake City UT USA

**Keywords:** DirectDensity, dual‐energy CT, image processing, imaging

## Abstract

**Purpose:**

Two new tools available in Radiation Oncology clinics are Dual‐energy CT (DECT) and Siemens’ DirectDensity™ (DD) reconstruction algorithm, which allows scans of any kV setting to use the same calibration. This study demonstrates why DD scans should not be used in combination with DECT and quantifies the magnitude of potential errors in image quality and dose.

**Methods:**

A CatPhan 504 phantom was scanned with a dual‐pass DECT and reconstructed with many different kernels, including several DD kernels. The HU values of various inserts were measured. The RANDO ^®^ man phantom was also scanned. Bone was contoured and then histograms of the bone HU values were analyzed for Filtered‐Backprojection (FBP) and DD reconstructions of the 80 and 140 kV scans, as well as several virtual, monoenergetic reconstructions generated from FBP and DD reconstructions. “Standard” dose distributions were calculated on several reconstructions of both phantoms for comparison.

**Results:**

The DD kernel overcorrected the high‐Z material inserts relative to bone, giving an excessively low relative electron density (RED). A unique artifact was observed in the high density inserts of the CatPhan in the monoenergetic scans when utilizing a DD kernel, due to the overcorrection in the DD scan of the material, especially at lower kV.

**Conclusions:**

While DD and DECT perform as expected when used independently, errors from their combined use were demonstrated. Dose errors from misuse of the DD kernel with DECT post‐processing were as large as 2.5%. The DECT post‐processing was without value because the HU differences between low and high energy were removed by the DD kernel. When using DD and DECT, we recommend the use of a DD reconstruction of the high energy scan for the dose calculation, and use of a FBP filter for the low and high energy scans for the DECT post‐processing.

## 1 INTRODUCTION

Multiple new technologies and imaging processing approaches have recently become available for CT Simulation. Two significant examples of this are DirectDensity™ (DD‐Siemens Medical) and Dual Energy CT (DECT). The vendor supplied white paper on DD states the following regarding the use of DD in combination with DECT: “It is technically possible to select DirectDensity™ kernels in reconstructions of Dual Energy scans, but the resulting DirectDensity™ images are not suitable for Dual Energy post‐processing.”^1^ It further states: “Non‐natural materials, for example metals and contrast agents like iodine, will decrease accuracy and — as with conventional CT images — can potentially lead to image artifacts.” However, the console allows DD kernels to be used on DECT scans. In the list of DECT algorithms on the Siemens Confidence CT 45 Simulator, there is a DD kernel, which could easily be selected without the user being aware of the potential problems involved. To our knowledge there has been no peer reviewed published data characterizing the various types of potential artifacts that may manifest when utilizing DD. We are also unaware of peer reviewed published data demonstrating the potential dosimetric consequences of performing dose calculations on scans using DD in combination with DECT. Here, we endeavor to explore and characterize both of these questions.

### 1.A Dual energy CT

In Hounsfield's first paper on CT, he discussed DECT and how various materials can be better differentiated by utilizing DECT post‐processing.^2^ The uses of DECT within the realm of Radiation Oncology are still a very active field of research.^3^ There are multiple hardware approaches to achieving DECT, each of which has its own strengths and weaknesses. One approach is a dual‐source system which has two x‐ray tubes 90° apart on the gantry. This approach is able to acquire the low and high energy scans simultaneously. Because it uses multiple tubes, it is able to use ideal mAs settings for the low energy tube and the high energy tube, and is capable of providing the best spectral differences of all the dual‐energy approaches. A dual‐source system is much more expensive due to a near doubling of the hardware involved.^4^, ^5^

Another approach is fast kV switching, which acquires low and high energy using the same tube in rapid succession, such that every other projection is high or low energy.^6^ This approach allows both low and high energy to be acquired in the same geometry and at approximately the same time. Because it uses one tube, the mAs is not ideal for both low and high energy.

Split filter is another approach which uses one tube and filtering of one half of the beam to create the high energy spectra. This approach allows both low and high energy to be acquired simultaneously. However, because the high energy is created solely by filtration, the spectral separation is not ideal. Another approach is to separate the low and high energy photons at the level of the detector.^5^, ^7^

Dual‐pass DECT uses one tube and two scans separated temporally. As such, it is the most susceptible to motion artifacts. However, it does allow for excellent spectral differences between low and high energy. This approach is also easiest to implement in existing clinics, as it requires no additional hardware.

There are many DECT post‐processing techniques which aid diagnostic clinical applications, such as metal artifact reduction, virtual noncontrast scans, material decomposition for higher Z materials relative to specific soft tissues^8^, ^9^ and virtual monoenergetic images.^10^ Virtual monoenergetic reconstructions are helpful in several ways. A low energy monoenergetic reconstruction provides much greater contrast. A high energy monoenergetic scan provides low noise and can be used to minimize metal artifacts. Often, the two are blended together to get the best contrast to noise ratio (CNR) for the best tumor visualization.

### 1.B CT sim

CT Simulation for radiation oncology imposes multiple demands, including an increased need for high spatial accuracy and HU accuracy, relative to diagnostic CT scanners.^11^, ^12^ The HU accuracy is important in Radiation Therapy because the CT scans’ HU values are converted to electron density which is then used to calculate the treatment dose.^13^ The CT‐to‐density conversion curve is energy dependent.^14^, ^15^ Radiation Oncology traditionally uses one standard energy (typically 120 kVp) at CT SIM because using multiple energies would require multiple CT‐density curves which is cumbersome in addition to the potential for misapplication of CT‐density curves.^16^

### 1.C DirectDensity™

Using one energy is not ideal for all patients or treatment sites. Lower energy scans provide much better soft‐tissue contrast. Patient size will also affect which energy is ideal. The larger the patient is, the higher the ideal scan energy to ensure sufficient signal. DirectDensity™ (DD) was developed by Siemens to address this issue of kV energy and CT‐density curves.^1^, ^17^ DD reconstructs and thresholds for bone; the bone is then forward projected into the sinogram space. The sinogram space representation is corrected for the length of bone along each ray path. Final reconstruction results in a CT that uses relative electron density (RED) units, not HU. This allows any beam energy to use the same CT‐density curve. Thus, the CT SIM software solution is capable of scanning with an ideal scan energy rather than always using the same kV settings.

However, one must be careful when acquiring images using DECT protocols and reconstructing the images using DD and performing DECT post‐processing. This study aims to quantify the potential image quality and dose implications of ignoring the vendor recommendations by performing DECT processing on DD images.

## 2 MATERIALS AND METHODS

To evaluate the effect of kernel selection on the resulting CT‐density curves, a dual energy scan of a CatPhan Model 504 was acquired on a SOMATOM Confidence^®^ RT Pro (Siemens Healthcare GmbH, Erlangen, Germany). The DECT was acquired using the DE_Abdomen_LiverVNC_late (Adult) protocol, with the Siemens default protocol settings. The 80 and 140 kVp scans were reconstructed with 24 different kernels, which differed according to the amount of smoothing, use of Sinogram Affirmed Iterative Reconstruction (SAFIRE), use of Iterative Metal Artifact Reduction (iMAR), and use of DD. Each of these kernel selections also have some DECT post‐processing to create a monoenergetic 40 keV and a monoenergetic 190 keV image. All 96 of these reconstructions were then analyzed for HU values (or RED values, when utilizing DD).

Multislice contours were created on each of the sensitometry inserts in the CTP404 module. The mean HU values within the contours were measured for each reconstruction. These were then plotted on a CT‐density curve and reviewed. Particular attention was paid to how the DD reconstructions handled the Teflon and Delrin inserts given the manufacturer warning that such materials could cause artifacts.^1^

To evaluate how well the DD algorithm handled bone, a RANDO^®^ man phantom (Rando) (The Phantom Laboratory, Greenwich, NY, USA) was used to perform additional scans. The same protocol was used for this as was used for the CatPhan. Because the focus was on the effect that DD had on DECT processing, only two kernels were used, one FBP kernel (B30f) and the DD kernel (E30f). These two data sets then had DECT post‐processing to create monoenergetic reconstructions at 40, 50, 70, 100, 120, 140, and 190 keV. A bone contour was created on the B30f 140 kVp scan (not the monoenergetic scan) using a threshold of 85 HU and above. That generated contour was evaluated to ensure that it accurately represented bone, and was then used to measure the average HU (or RED) of the bone in all other reconstructions.

Additional monoenergetic reconstructions at 50, 70, 100, 120, and 140 keV were created from the CatPhan scan, on the same kernels as those used for Rando (B30f and E30f). The Teflon insert average HU (or RED) values were compared with the bone contour measurements from Rando to examine the differences between bone and high‐Z non‐natural‐body materials.

To evaluate potential dosimetric impacts from performing DECT post‐processing on DD scans and utilizing the standard DD CT‐density curve, a treatment plan was created on the CatPhan scans. The plan was comprised of two 18X lateral opposed beams each delivering 100 MU. This was performed on Eclipse 13.5 utilizing AAA External Beam 13.5.35 (Varian Medical Systems, Palo Alto, CA, USA). An additional plan was created on the Rando phantom. The plan was a T7 SBRT Spine plan utilizing 6XFFF arcs.

## 3 RESULTS

**Figure 1:**
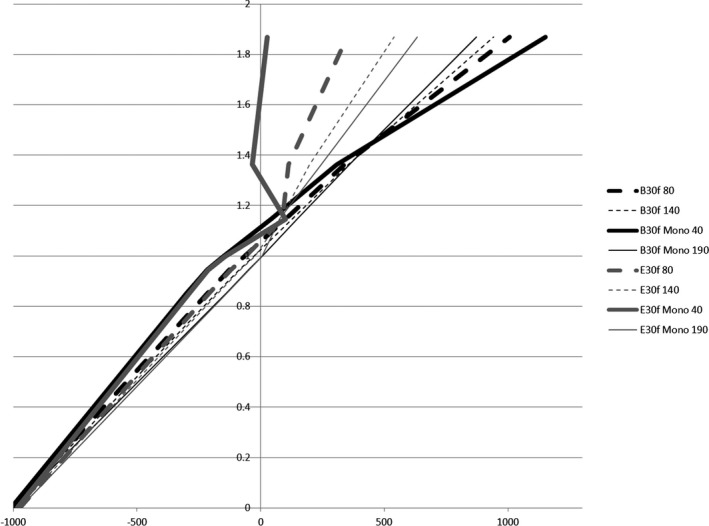
CT‐Density curves for a high and low energy scan, 40 and 190 keV monoenergetic scans, all of which reconstructed with a FBP kernel (B30f) and a DD kernel (E30f).

**Figure 2:**
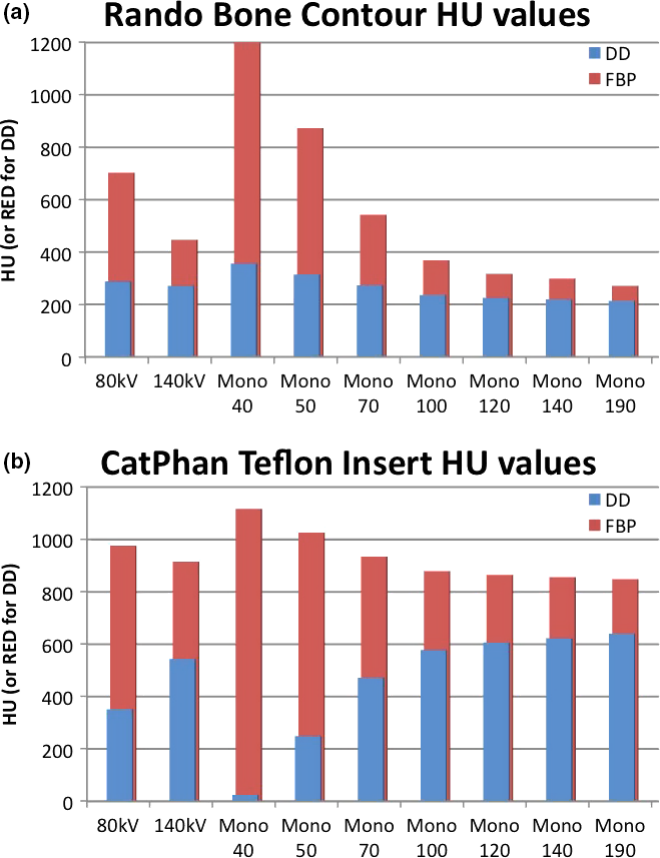
Non‐natural‐body material vs natural material performance: A comparison of the mean HU values of the bone contour of the Rando phantom (a) and the Teflon insert in the CatPhan (b). The low and high energy scans along with seven different virtual monoenergetic scans are shown. The DD overcorrects the HU of Teflon, inverting the typical ratio. This is what leads to significantly different extrapolations in the monoenergetic reconstructions.

**Figure 3:**
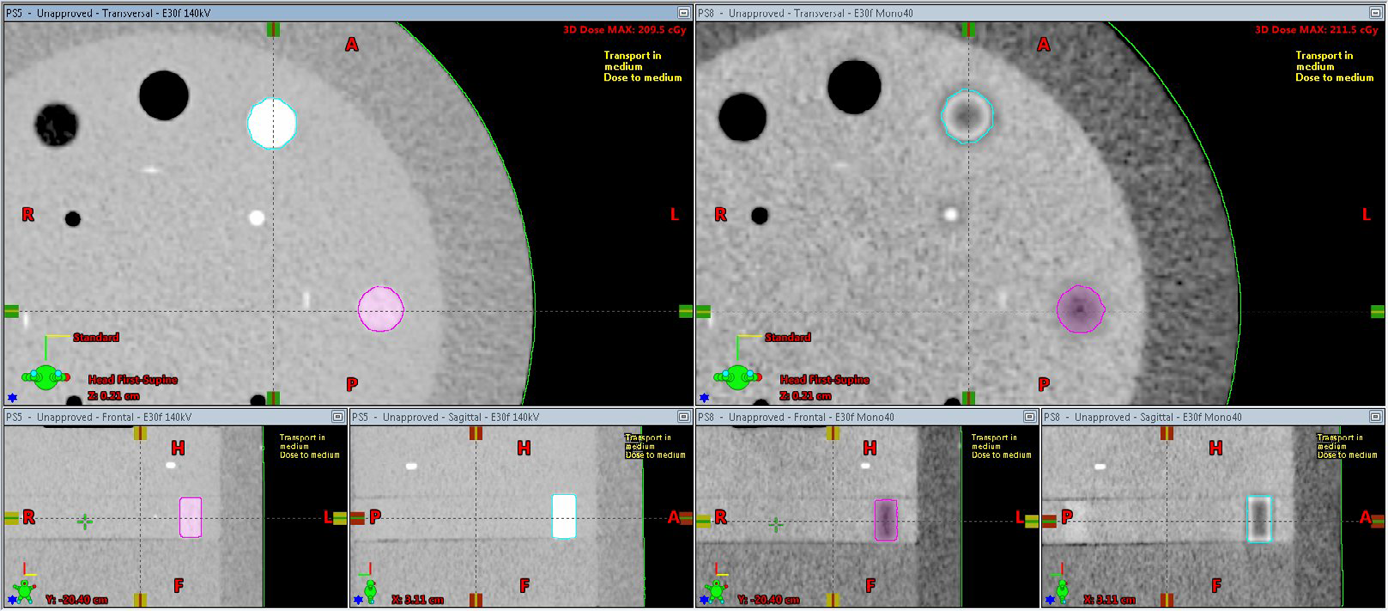
The DD 140 kV reconstruction of the CatPhan on the left, next to the DD monoenergetic 40 keV reconstruction on the right. DECT post‐processing performed on DD reconstructions gave rise to the unique artifacts seen on the right in the high density inserts

**Figure 4:**
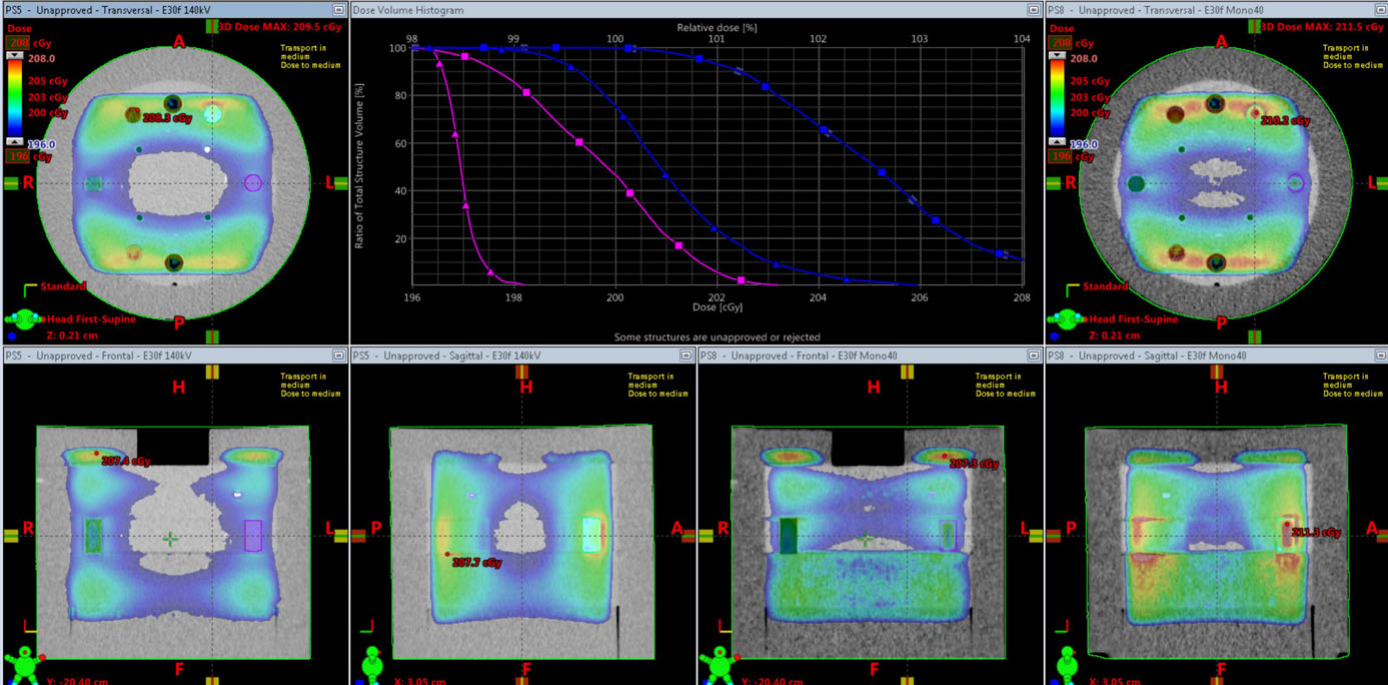
Dose from lateral opposed 18X beams on a CatPhan. The color wash and DVH window have a minimum of 196 cGy and a maximum of 208 cGy. The blue contour is the Teflon insert and the pink is the Delrin insert. The orthogonal views on the left are the 140 kV DD reconstruction and are represented on the graph by the square curves. The orthogonal views on the right are the 40 keV monoenergetic DD reconstruction and are represented on the graph by the triangle curves.

**Figure 5:**
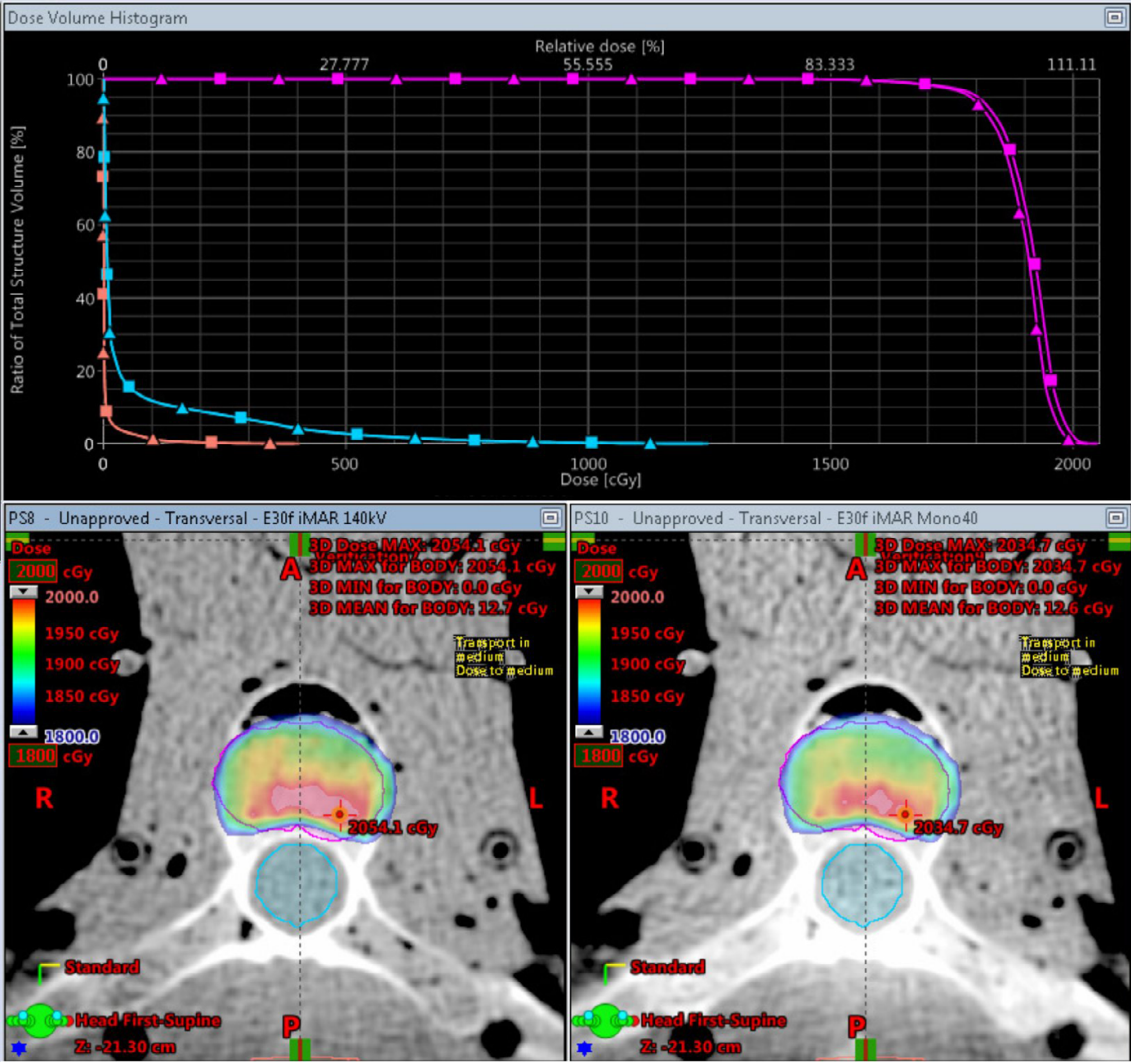
Dose from an SBRT spine plan on Rando. The magenta contour is the PTV, the blue contour is the spine, and the peach contour is the skin. The view on the bottom left is the 140 kVp DD reconstruction and is represented on the graph by the square curves. The view on the bottom right is the 40 keV monoenergetic DD reconstruction and is represented on the graph by the triangle curves.

The DD algorithm removes the differences in high HUs between scans of different energies as it converts HU to RED. DECT utilizes differences in HUs between scans of different energies to calculate the virtual HU values of monoenergetic scans. DECT post‐processing on any DD reconstructions results in meaningless results (Fig. 1). The HU values for the monoenergetic 190 keV reconstructions can be viewed as a reasonable estimate of the RED because the photon attenuation is more dominated by Compton scatter. As can be seen in Fig. 2(a), the 271 HU value of the monoenergetic 190 keV scan is the same as the DD corrected 271 RED value from the 140 kV scan. The DD algorithm overcorrected the high density inserts in the CatPhan relative to the FBP monoenergetic 190 keV values [Fig. 2(b)]. This overcorrection led to a lower RED for the low energy than the RED for the high energy. Unique artifacts, in which the edge of a high density object has decreased CT‐values and the center of the high density object has decreased CT‐values, were generated in the CT by the combination of DD overcorrection and DECT post‐processing on the DD scans (Fig. 3). In contrast to that behavior, the DD algorithm performed well on bone, bringing the RED values for the low and high energy scans into relatively close agreement [Fig. 2(a)]. Dose calculated on the CatPhan with a 40 keV monoenergetic reconstruction of DD scans resulted in a maximum dose difference of 2.5% relative to the 140 kVp DD scan (Fig. 4). Dose calculated on Rando with a 40 keV monoenergetic reconstruction of DD scans resulted in a maximum dose difference of less than 1% relative to the 140 kVp DD scan (Fig. 5).

Figure 4 shows the dose differences between a 140 kVp DD scan and a virtual monoenergetic 40 keV DD reconstruction. The color wash is bounded by the same level that the *x*‐axis of the DVH is set, with a minimum value of 196 cGy and a maximum value of 208 cGy. The Teflon and Delrin inserts saw dose differences as much as 2.5%. Considering the shape of the CT‐density curve for the 40 keV DD reconstruction (Fig. 1) this dose difference is quite modest.

Figure 5 shows the dose differences between a 140 kVp DD scan and a virtual monoenergetic 40 keV DD reconstruction. The color wash is bounded from 1800 to 2000 cGy. There were functionally no differences in dose to the spine or patient skin contours. The dose to the PTV saw some slight differences, with the 40 keV monoenergetic reconstruction seeing a slightly less than 1% decrease in dose.

## 4 DISCUSSION

The principles upon which DD works and derives its advantages are the reasons that DD cannot be used with DECT. By converting the HU values of high density materials from scans of various energies into RED values, DD removes the differences that DECT utilizes. When the HU (or RED) values of the CatPhan inserts were measured (on the 96 reconstructions utilizing 24 different kernels and DECT post‐processing) and plotted, they fell into eight distinct groups of overlapping CT‐density curves. For ease of viewing, one curve from each group was selected for inclusion in Fig. 1. On the left half of the graph, there are four groups of curves, one for the low energy scan, one for the high energy scan, and one each for the 40 and 190 monoenergetic reconstructions. This was the case regardless of the use of DD or FBP. On the right half of the graph, the curves do not fall into neat groups although the DD curves are clearly diverging from the FBP curves. In Fig. 1, the black curves are typical FBP kernel reconstructions. The dashed curves are the high and low energy scans while the solid lines are the virtual monoenergetic reconstructions. These FBP reconstructions show the typical shifts on the CT‐density curve that we expect to see with DECT: high density objects have a higher HU on lower energy scans, and low density objects have a lower HU on lower energy scans. The DD scans closely follow the FBP scans for low density objects. This makes sense because the DD algorithm thresholds for bone and then in the conversion to RED from HU it will primarily adjust those voxels, not the low HU voxels. At higher densities with non‐natural‐body materials, we see the DD algorithm overcorrect the values. Siemens alerts users that non‐natural‐body materials, like the high density inserts in the CatPhan, will have decreased accuracy and could lead to artifacts. This overcorrection inverts the now RED values for high density objects relative to their FBP HU values. The DECT processing then accentuates this overcorrection for low energy monoenergetic reconstructions. This is particularly apparent when looking at the Teflon HU/RED values compared to Bone HU/RED values (Fig. 2). This is also what gave rise to these unique image artifacts seen in the high density inserts in Fig. 3. It should also be noted that while this study only saw an overcorrection by DD of non‐natural‐body dense objects, there is the potential for the DD algorithm to under‐correct for other materials.

The Rando phantom was also scanned and reconstructed with DD kernels and then had DECT post‐processing. The unique artifacts visible on the CatPhan (Fig. 3) were not visible on any of these reconstructions. As shown in Fig. 2(a), the bone was not overcorrected by DD which is what had led to the artifact on the CatPhan. For these artifacts to occur there must be DECT post‐processing of DD reconstructed images with non‐natural‐body materials.

To more closely model clinically relevant dose differences, the spine SBRT plan was created on Rando and calculated on a 140 kVp DD scan and a virtual monoenergetic 40 keV DD reconstruction. As noted in the results, there was less than 1% difference in dose to the PTV. For the cases explored here, where a clinic might accidentally/inadvertently ignore multiple vendor recommendations, the observed dosimetric error was seen to be relatively small for photon planning.

Given the impacts on dose and image quality, the biggest concern for a clinic that utilizes DD with DECT post‐processing appears to be the potential for inaccurate contouring due to artifacts from non‐natural‐body materials such as iodine contrast or metal implants. We note that while dose calculation errors can be certainly problematic, errors in contouring of the target, for instance, can lead to target misses which are, of course, 100% errors. Should a clinic want to utilize both DECT and DD, there are multiple ways to do so while also heeding vendor recommendations. The ease of using DD is the ability to use any energy and still only worry about one CT‐density curve. This can still be accomplished by creating one reconstruction using DD for the dose calculation, while using FBP kernels for the DECT reconstructions, which could provide better image quality for more accurate contouring. If this is being done with a DECT protocol, we would recommend using the DECT option of generating a DE Rho image (which decomposes the values into a Z component and a RED component). The RED component could then utilize the DD CT‐density curve.

## 5 CONCLUSIONS

DirectDensity™ and DECT are both promising technologies for CT SIM in Radiation Oncology. DD will allow CT Simulation with an optimal beam energy for the best image quality while avoiding potential misapplication of CT‐density curves. DECT can provide improved imaging, including virtual monoenergetic reconstructions. DECT can improve contouring accuracy. Clinics should heed vendor recommendations about their use. If ignored, unique DD/DECT artifacts can occur. Knowledge of this is particularly important while there is a DD kernel listed in a group of DECT kernels. While the dosimetric impact observed for cases explored here was relatively small, subsequent artifacts from non‐natural‐body materials could lead to inaccurate target and OAR delineation, which could subsequently lead to more significant dose distribution errors. If clinics wish to utilize both technologies simultaneously, the DECT processing should only be performed with FBP kernels. The DD reconstruction should be created using DE Rho post‐processing to minimize artifacts from non‐natural‐body materials.

## CONFLICT OF INTEREST

There are no conflicts of interest to report.

## References

[1] Ritter A , Mistry N . DirectDensity™: Technical principles and implications for radiotherapy – white paper. Tech. Rep. (Erlangen: Siemens Healthineers, 2016).

[2] Hounsfield GN . Computerized transverse axial scanning (tomography): part 1. description of system. Br J Radiol. 1973;46:1016–1022. 4757352 10.1259/0007-1285-46-552-1016

[3] van Elmpt W , Landry G , Das M , Verhaegen F . Dual energy CT in radiotherapy: current applications and future outlook. Radiother Oncol. 2016;119:137–144. 26975241 10.1016/j.radonc.2016.02.026

[4] Flohr TG , Leng S , Yu L , et al. Dual‐source spiral CT with pitch up to 3.2 and 75 ms temporal resolution: image reconstruction and assessment of image quality. Med Phys. 2009;36:5641–5653. 20095277 10.1118/1.3259739

[5] Almeida IP , Schyns LE , Öllers MC , et al. Dual‐energy CT quantitative imaging: a comparison study between twin‐beam and dual‐source CT scanners. Med Phys. 2017;44:171–179. 28070917 10.1002/mp.12000

[6] Goodsitt MM , Christodoulou EG , Larson SC . Accuracies of the synthesized monochromatic CT numbers and effective atomic numbers obtained with a rapid kVp switching dual energy CT scanner. Med Phys. 2011;38:2222–2232. 21626956 10.1118/1.3567509

[7] Euler A , Parakh A , Falkowski AL , et al. Initial results of a single‐source dual‐energy computed tomography technique using a split‐filter: assessment of image quality, radiation dose, and accuracy of dual‐energy applications in an in vitro and in vivo study. Invest Radiol. 2016;51:491–498. 26895193 10.1097/RLI.0000000000000257

[8] Bazalova M , Carrier J‐F , Beaulieu L , Verhaegen F . Dual‐energy CT‐based material extraction for tissue segmentation in Monte Carlo dose calculations. Phys Med Biol. 2008;53:2439. 18421124 10.1088/0031-9155/53/9/015

[9] Lehmann L , Alvarez R , Macovski A , et al. Generalized image combinations in dual kVp digital radiography. Med Phys. 1981;8:659–667. 7290019 10.1118/1.595025

[10] Yu L , Leng S , McCollough CH . Dual‐energy CT–based monochromatic imaging. Am J Roentgenol. 2012;199:S9–S15. 23097173 10.2214/AJR.12.9121

[11] Groell R , Rienmueller R , Schaffler G , Portugaller H , Graif E , Willfurth P . Ct number variations due to different image acquisition and reconstruction parameters: a thorax phantom study. Comput Med Imaging Graph. 2000;24:53–58. 10767584 10.1016/s0895-6111(99)00043-9

[12] Kilby W , Sage J , Rabett V . Tolerance levels for quality assurance of electron density values generated from CT in radiotherapy treatment planning. Phys Med Biol. 2002;47:1485. 12043814 10.1088/0031-9155/47/9/304

[13] Cozzi L , Fogliata A , Buffa F , Bieri S . Dosimetric impact of computed tomography calibration on a commercial treatment planning system for external radiation therapy. Radiother Oncol. 1998;48:335–338. 9925254 10.1016/s0167-8140(98)00072-3

[14] Constantinou C , Harrington JC , DeWerd LA . An electron density calibration phantom for CT‐based treatment planning computers. Med Phys. 1992;19:325–327. 1584125 10.1118/1.596862

[15] Watanabe Y . Derivation of linear attenuation coefficients from CT numbers for low‐energy photons. Phys Med Biol. 1999;44:2201. 10495115 10.1088/0031-9155/44/9/308

[16] Nobah A , Moftah B , Tomic N , Devic S . Influence of electron density spatial distribution and x‐ray beam quality during CT simulation on dose calculation accuracy. J Appl Clin Med Phys. 2011;12:80–89. 10.1120/jacmp.v12i3.3432 PMC5718643 21844854

[17] van der Heyden B , Öllers M , Ritter A , Verhaegen F , van Elmpt W . Clinical evaluation of a novel CT image reconstruction algorithm for direct dose calculations. Phys Imaging Radiat Oncol. 2017;2:16.

